# Neuroimmune Dysregulation and Synaptic Pruning in Autism Spectrum Disorder

**DOI:** 10.3390/biology15171539

**Published:** 2026-09-04

**Authors:** Abdel Bernal-Reyes, Ormany Soriano-Torres, Iris Dany Carmenate Rodríguez, Deanira Patrone, Nicola Antonucci, Dario Siniscalco, Maria de los Angeles Robinson-Agramonte

**Affiliations:** 1 Department of Immunology, Instituto de Ciencias Básicas y Preclínicas “Victoria de Girón”, Universidad de Ciencias Médicas de La Habana, La Habana 10600, Cuba; bernalabdel962@gmail.com (A.B.-R.); ormany87@gmail.com (O.S.-T.); 2 Hospital Pediátrico Provincial “Docente José Martí”, Sancti Spiritus 60100, Cuba; irisdany18@gmail.com; 3 Department of Experimental Medicine, Division of Biotechnology, Molecular Biology and Histology, University of Campania “L. Vanvitelli”, 80138 Naples, Italy; deanirantonietta.patrone@unicampania.it (D.P.); dario.siniscalco@unicampania.it (D.S.); 4 Biomedical Centre for Autism Research and Treatment, 70126 Bari, Italy; info@antonucci.eu; 5 International Center for Neurological Restoration, La Habana 11300, Cuba

**Keywords:** autism spectrum disorder, synaptic pruning, neuroinflammation, neuroimmune dysregulation, synaptic plasticity

## Abstract

Autism spectrum disorder (ASD) is a neurodevelopmental condition characterized by social communication deficits and repetitive behaviors, now affecting approximately 1 in 31 children. While traditionally defined behaviorally, ASD is increasingly understood as a disorder of brain connectivity arising from altered synaptic formation and refinement. This narrative review synthesizes evidence on neuroimmune dysregulation in ASD, focusing on immune-mediated synaptic pruning mechanisms. We conducted a comprehensive literature search in PubMed, Scopus, and Web of Science (2010–2026), prioritizing high-impact peer-reviewed research. Convergent findings suggest that the classical complement cascade (*C1q*-*C3*) tags specific synapses for elimination, while microglia participate in the phagocytic removal of tagged connections. Genetic studies have reported associations between ASD and variants in complement-related genes (*C1q*, *C3*, *CR3*, and *C4A*, although the strongest evidence for *C4A*-mediated pruning comes from schizophrenia research), as well as in microglial function genes (*TREM2*, *PTEN*, *SHANK3*). Neuroimaging reveals a dynamic pattern of local hyperconnectivity transitioning to long-range hypoconnectivity during development, particularly affecting prefrontal, insular, and cerebellar regions. Systemic inflammation, including gut–brain axis dysbiosis and maternal immune activation, may amplify neuroimmune dysregulation. We conclude that ASD can be understood, in part, as a disorder of synaptic immunology, where disrupted neuroimmune communication during critical developmental windows may contribute to altered connectivity. The complement–microglia axis therefore represents a potential mechanistic target for future therapeutic investigation.

## Simple Summary

Autism spectrum disorder (ASD) affects millions of children and adults worldwide, yet its underlying causes remain incompletely understood. This review examines a growing body of evidence suggesting that the brain’s immune system—long thought to be isolated from the rest of the body—plays a critical role in shaping how neural connections are formed and refined during development. During early life, the brain produces an excess of connections between nerve cells, and those that are not strengthened through experience are normally eliminated through a process called “synaptic pruning.” This pruning process is orchestrated by immune molecules and specialized brain-resident immune cells called microglia. In ASD, this pruning mechanism appears to be disrupted, leading to either too many or improperly organized connections that may contribute to the characteristic features of the condition, including difficulties with social communication and sensory processing. The review also explores how inflammation elsewhere in the body can influence brain immune function. Understanding these mechanisms opens new possibilities for developing treatments that target the underlying biological processes rather than just managing symptoms.

## 1. Introduction

Autism spectrum disorder (ASD) is a neurodevelopmental condition characterized by persistent deficits in communication and social behavior and interests [1]. Classically defined by its behavioral manifestations, ASD has been reconceptualized as a disorder of brain connectivity where alterations in the formation, refinement, and stabilization of synapses underlie its clinical phenotypes [2].

Historically, the central nervous system (CNS) was considered an immunologically privileged site, a concept that suggested relative isolation from peripheral immune surveillance [3]. However, subsequent discoveries have refined this paradigm, indicating that the CNS is not absolutely isolated from immune surveillance, but instead possesses specialized and highly regulated mechanisms of immune communication and surveillance. The identification of meningeal lymphatic vessels, the characterization of the glymphatic system, and the understanding of the active role of resident cells such as microglia and astrocytes have redefined CNS immunology [4,5]. This constant dialogue between the nervous system and the immune system is not only crucial for maintaining homeostasis but also plays a fundamental role in neural development processes, including synaptogenesis and synaptic pruning [6,7].

Synaptic pruning is an essential process through which redundant or weakly active synaptic connections are selectively eliminated, thereby allowing the optimization of neural circuits during critical developmental windows [8]. This has been shown not to be a passive process but rather one directed by precise immunological mechanisms, in particular the complement system—specifically the opsonins *C1q* and *C3*—and microglial cells, which act in a coordinated manner to tag and phagocytose synapses, in a process molecularly analogous to pathogen elimination in the periphery [9].

Dysregulation of this neuroimmune axis has emerged as a potential pathophysiological mechanism that may contribute to ASD. Large-scale genomic studies have associated variants in complement system genes, such as *C4A* genes, with an increased risk of neurodevelopmental disorders, suggesting a mechanism of aberrant synaptic pruning [10,11]. Furthermore, atypical microglial activation and states of chronic low-grade neuroinflammation have been reported in individuals with ASD and may influence mechanisms involved in the balance between synapse formation and elimination [11,12]. This inflammation can have diverse origins, including maternal immune activation (MIA), gut–brain axis dysbiosis, and autoimmune responses [13].

This narrative review synthesizes the most current evidence on neuroimmune dysregulation, with emphasis on immune mediated synaptic pruning mechanisms, the etiology, and the pathophysiology of ASD. We propose that ASD can be understood, in part, within a framework of “synaptic immunology,” in which alterations in neuroimmune communication during critical developmental windows may contribute to atypical synaptic refinement and brain connectivity. Finally, we explore the translational implications of this framework, highlighting the complement microglia axis as a promising target for the development of innovative therapeutic interventions.

## 2. Methods

### 2.1. Search Strategy

For this narrative review, we searched Medline/PubMed, Google Scholar and Web of Science. In all cases, the searches were conducted for papers in the English language, published from 1 January 2010 to 31 March 2026, using MESH terms and free text words for the following search keys: (“autism spectrum disorder” OR ASD) AND (“synaptic pruning” OR “microglia” OR “complement” OR “neuroinflammation” OR “immune dysregulation”).

### 2.2. Inclusion and Exclusion Criteria

We reviewed all of the articles published in the English language and selected the most relevant ones, excluding those not written in English, conference abstracts/not-indexed papers, opinion articles, and studies that did not directly address neuroimmune mechanisms in ASD. Papers were searched, and only articles in English were found.

### 2.3. Data Collection and Extraction

Firstly, two authors searched and screened the Medline/PubMed, Google Scholar, and Web of Science databases for relevant titles and abstracts. Full text articles and related references were subsequently searched and analyzed. Debates on inclusion/exclusion criteria were resolved by team consensus. Articles published on Neuroimmune dysregulation and Synaptic pruning in ASD were selected, original and review papers were included.

## 3. Neuroimmunology of the Developing CNS

The conception of the CNS as an immunologically privileged organ has been revised in recent decades. Rather than being an immunologically privileged site, the CNS constitutes a dynamic ecosystem where the nervous system and the immune system establish a constant dialogue, highlighting its essential role in both defense and neural development physiology [14,15]. This dialogue is mediated by specialized anatomical structures, resident cell populations, and an active exchange with peripheral immunity, which collectively regulate homeostasis, plasticity, and brain maturation.

### 3.1. Specialized Barriers and Neuroimmune Communication

The CNS is protected by an integrated set of barriers that selectively regulate the traffic of molecules and immune cells between the systemic compartment and the nervous system: the blood–brain barrier (BBB), the blood cerebrospinal fluid (CSF) barrier, and the meninges. The BBB, composed of tightly joined endothelial cells, pericytes, and astrocytic end feet, acts as a highly selective filter [16]. During development, the BBB not only restricts passage but also mediates the active transport of cytokines and growth factors crucial for neurogenesis and synaptogenesis [4].

The revolutionary discovery of functional meningeal lymphatic vessels provided new arguments against the dogma of no immunological response in the CNS due to the absence of lymphatic drainage [5]. This network, together with the glymphatic system (an aquaporin 4 dependent clearance mechanism in astrocytes), facilitates the removal of metabolic waste and the presentation of CNS antigens to deep cervical lymph nodes [6,7]. This circuit constitutes an anatomical pathway for immune surveillance and modulation of inflammatory responses, playing a potential role in the controlled elimination of synaptic components during development.

### 3.2. Microglia as Developmental Modulator

Glial cells, particularly microglia and astrocytes, are central actors in developmental neuroimmunology. Microglia, derived from yolk sac progenitors, colonize the CNS during embryogenesis and constitute the main resident immune cell population [17]. Beyond their role in defense, microglia perform fundamental functions in neurogenesis, axonal guidance, synaptic surveillance, and synaptic pruning [9,18]. Through their dynamic processes, they establish transient contacts with synapses, monitoring their activity and functional state.

### 3.3. The Complement System in the Developing CNS

A paradigmatic finding of neuroimmune synaptic plasticity was the identification of molecular components involved in the restricted exchange of molecules across the barrier system for the maintenance of internal brain homeostasis. A component little addressed in this context is the complement cascade of the innate immune system. This system, classically associated with pathogen opsonization, was phylogenetically “co opted” to serve as a molecular signaling mechanism that tags specific synapses for elimination. Complement components such as *C1q* and *C3* are expressed in neurons and synapses during critical postnatal developmental windows [12]. Likewise, the classical complement pathway (*C1q C3*) is activated in weakly active or redundant synapses, depositing the opsonizing fragment *C3b*, which is recognized by complement receptors (e.g., CR3) expressed on microglia, facilitating phagocytosis of the tagged synapse [13]. From this approach, it follows that this process is fundamental for circuit refinement in regions such as the visual cortex, hippocampus, and prefrontal cortex.

### 3.4. Neuroimmune Imbalance, Neurodevelopment, and Autism Spectrum Disorder

Neuroimmune imbalance during sensitive developmental periods can have profound consequences for brain connectivity. In the context of ASD, converging evidence points to a dysregulation of these processes involving cellular and humoral immune elements:

Microglia: Post-mortem and neuroimaging studies have reported, for example, atypical microglial activation and increased microglial density in cortical and cerebellar regions of individuals with ASD [19,20]. Animal models of ASD also show dysfunctional microglial phenotypes, with altered phagocytic capacity and pro-inflammatory cytokine secretion profiles that argue for the role of these cells in the aberrant connectivity that occurs in this entity [21].

Complement System: Similarly, genomic studies have suggested a possible involvement of these complement system proteins in aberrant synaptic pruning mechanisms. It is known that polymorphisms and variants in complement system genes, such as *C4A* and *C3*, have been associated with an increased risk of ASD [10,22].

Systemic inflammation and neural immunity: Maternal immune activation (MIA) during gestation, an established environmental risk factor for ASD, induces a persistent fetal neuroinflammatory state, altering microglial function and complement component expression, which can lead to defects in synaptic refinement [23,24].

Recent evidence further supports a framework in which peripheral immune perturbations may influence neuroimmune signaling during development. The Peripheral-to-Central Inflammatory Cascade-Amplification Model (PC-ICAM) proposes a mechanistic continuum linking maternal immune activation (MIA) during gestation with the transmission of inflammatory signals to the fetal brain and their subsequent amplification within the central nervous system. Within this framework, IL-6 and IL-17 have been implicated as important mediators of MIA-related effects, together with signaling pathways including NF-κB, JAK/STAT, MAPK/ERK, NLRP3, and PI3K-AKT-mTOR, which may influence synaptic homeostasis, microglial function, and excitation-inhibition balance [25]. An additional mechanistic pathway involves the purinergic P2X7 receptor/NLRP3 inflammasome/IL-1β axis. Preclinical evidence indicates that activation of this pathway in the context of maternal immune challenge can increase IL-1β signaling and affect neurodevelopmental processes, including neuronal migration, dendritic arborization, synapse formation, and blood–brain barrier integrity. Pharmacological modulation of the P2X7/NLRP3/IL-1β cascade has also been reported to mitigate adverse neurodevelopmental outcomes in experimental models, suggesting that this pathway warrants further investigation as a potential mediator of MIA-associated neurodevelopmental alterations [26]. The gut microbiota represents another potential modulator of neuroimmune homeostasis. Alterations in gut microbial composition and microbial metabolites, including short-chain fatty acids (SCFAs), have been associated with changes in peripheral immune signaling and microglial maturation and function. Proposed mechanisms include modulation of GPR43/GPR109A-related signaling and histone deacetylase activity, as well as changes in circulating pro-inflammatory mediators such as IL-6, IL-17A, and TNF-α that may communicate with the CNS through humoral, neural, and blood–brain barrier-associated routes. Gut-derived signals may also influence peripheral Th17 responses, providing a potential link between maternal or systemic immune activation and neurodevelopmental alterations [27]. These gut-immune-brain interactions may involve several interconnected pathways. Experimental evidence suggests that alterations in the gut microbiota can influence the indole-3-propionic acid/AHR/NF-κB axis and microglial activity, while microbial products and metabolites may affect blood–brain barrier integrity and astrocytic function. These interactions may also be bidirectional, because changes in central neuroimmune signaling can potentially influence gastrointestinal function through autonomic and neuroendocrine pathways [28]. Collectively, these findings provide biologically plausible mechanisms through which peripheral immune and microbial signals may interact with central neuroimmune processes relevant to synaptic development.

Nevertheless, the strength of evidence differs substantially across experimental systems. A physiological role for microglia and complement signaling in synaptic remodeling during CNS development is well supported, and human post-mortem and neuroimaging studies have reported alterations in microglial states and density in individuals with ASD. In contrast, direct mechanistic links among maternal immune activation, gut dysbiosis, complement dysregulation, microglial dysfunction, and aberrant synaptic pruning derive predominantly from animal and other preclinical models. Although these findings are broadly consistent with a potential neuroimmune contribution to ASD-related neurodevelopmental alterations, heterogeneity in study populations, experimental models, developmental timing, and methodological approaches limits direct comparison and causal inference. Therefore, current evidence supports these pathways as biologically plausible contributors to ASD in at least a subset of individuals rather than demonstrating a uniform causal mechanism across the autism spectrum.

## 4. Synaptic Pruning: Molecular Mechanisms and Relevance for ASD

Synaptic pruning is a fundamental neurodevelopmental process that favors the selective elimination of redundant or weakly active synaptic connections, allowing the refinement and optimization of neural circuits [6,29]. This process, regulated in part by highly conserved immune-related mechanisms, is essential for the formation and refinement of efficient neural networks and may be altered in ASD, potentially contributing to atypical patterns of brain connectivity [30].

### 4.1. The Complement System as a Molecular Marker in Synaptic Pruning

The finding that components of the complement system participate in CNS development revolutionized the understanding of synaptic pruning. Thus, it has been shown that complement proteins such as *C1q*, initiator of the classical pathway, and the central effector *C3*, are expressed in the postnatal CNS and are specifically localized at synapses [6,29]. In this context, the mechanism of action by which the classical complement pathway acts in weakly active synapses consists of *C1q* depositing and activating the complement cascade, culminating in the cleavage of *C3* and the covalent deposition of its *C3b*/*iC3b* fragment on the synaptic membrane. This fragment acts as a potent opsonin, i.e., an “eat me” molecular signal [30,31].

For its part, microglia, the main phagocytic cell resident in the CNS, expresses complement receptor 3 (CR3), which specifically recognizes *C3b*/*iC3b*. Binding triggers phagocytosis of the tagged synapse, physically eliminating it from the circuit [29,30]. Deficiencies in this axis can result in altered synaptic refinement and connectivity in experimental models. Studies using *C1q*-, *C3*-, or CR3-deficient mice have demonstrated persistent defects in synaptic elimination, providing important preclinical evidence for the potential relevance of complement-dependent pruning mechanisms to neurodevelopmental conditions, including ASD [6,29].

### 4.2. Additional Molecular Signals in Synaptic Selection

Molecular signals in synaptic selection involve pro-phagocytic and anti-phagocytic signals. Research has identified a molecular dialogue that determines the fate of each synapse, thus forming a network of signals involving phagocytic/anti phagocytic and extrinsic and intrinsic mechanisms, which ensure that pruning is a balanced, precise, and highly selective process, monitored by a very complex signaling system, beyond simple tagging of connections for elimination:

Pro-phagocytic (“Eat Me”) Signals:

Phosphatidylserine (PS): This is a phospholipid normally confined to the inner leaflet of the plasma membrane. During critical developmental periods, PS is locally exposed on the surface of dendritic spines and healthy but destined for elimination synapses, an exposure that serves as a direct “eat me” signal recognized by microglial receptors such as *TREM2*, guiding phagocytosis [31].

Lack of neuronal activity: Synaptic activity modulates susceptibility to complement tagging. Synapses with low neuronal activity are more prone to *C1q* deposition and, therefore, to elimination [29].

Anti-phagocytic (“Don’t Eat Me”) Signals:

Extrinsic mechanism: CD47 SIRPα: The CD47 molecule, expressed on the synaptic surface, interacts with its receptor SIRPα on microglia, transmitting an inhibitory signal that protects the synapse from phagocytosis. This system acts as a crucial counterbalance to the complement system, safeguarding critical connections during these neurodevelopmental windows [29].

Intrinsic mechanism: Endogenous inhibitors, proteins such as SRPX2, secreted by neurons, can bind to *C1q* and locally inhibit the activation of the complement cascade, providing another level of regulation to prevent excessive pruning [30].

This signaling network ensures that pruning is a balanced and highly selective process.

### 4.3. Implications of Aberrant Synaptic Pruning for Autism Spectrum Disorder

Dysregulation of immune-mediated pruning mechanisms represents a plausible pathophysiological hypothesis for ASD. Available evidence suggests that altered pruning, potentially including insufficient pruning in some contexts, may contribute to patterns of cortical and cerebellar hyperconnectivity reported in ASD [30,32].

In the context of the complement genetics, although the strongest association between complement genetic variants and neurodevelopmental disorders has been established for schizophrenia (with the *C4A* gene), complement genes are of great translational relevance for ASD [29]. Moreover, polymorphisms in complement system genes (*C1q*, *C3*, *C4*) and their regulators could alter the efficiency or specificity of synaptic tagging, potentially predisposing to aberrant circuit refinement [30].

Atypical microglial activation has been repeatedly reported in ASD; however, these observations should not be considered equivalent to direct evidence of microglial dysfunction or impaired synaptic pruning. Alterations in microglial phagocytic capacity, whether associated with chronic reactive states or hyporeactive phenotypes, could potentially interfere with complement- and activity-dependent synaptic pruning [33]. Such alterations may arise in association with genetic or environmental factors, including maternal immune activation (MIA) [33].

Consequently, insufficient pruning during critical developmental windows, particularly in higher-order association regions such as the prefrontal cortex, could contribute to the retention of redundant synaptic connections. Such alterations have been proposed as one possible mechanism underlying patterns of hyperconnectivity and associated features such as altered sensory processing, difficulties in social information integration, and repetitive behaviors in ASD [30].

Taken together, the evidence reviewed in this section establishes a well-supported physiological role for complement signaling, microglial phagocytosis, and additional pro- and anti-phagocytic signals in the regulation of synaptic pruning. However, their specific contribution to aberrant synaptic pruning in ASD is less firmly established. Direct mechanistic evidence for complement- and microglia-dependent synapse elimination derives predominantly from experimental and animal studies, whereas human ASD studies provide mainly genetic, post-mortem, and neuroimaging evidence that is consistent with, but does not directly demonstrate, altered pruning. Moreover, current findings do not necessarily support a uniform direction of pruning abnormalities across ASD, and differences in developmental stage, brain region, genetic background, and experimental model may contribute to divergent observations. Thus, current evidence supports aberrant immune-mediated synaptic pruning as a biologically plausible mechanism that may contribute to altered neural connectivity in subsets of individuals with ASD, while a direct causal relationship in humans remains to be established.

## 5. Alterations in Synaptic Pruning in ASD: Genetic and Molecular Evidence

The hypothesis that dysregulation of immune mediated mechanisms may contribute to altered synaptic pruning in ASD is supported by converging, although heterogeneous, lines of evidence, including post-mortem brain tissue analyses, animal models, and genomic studies. Together, these findings provide a framework for investigating potential relationships among genetic risk, neuroimmune dysregulation, synaptic refinement, and altered brain connectivity.

### 5.1. Genetic Evidence

Genome-wide association studies (GWAS) and copy number variation (CNV) analyses have repeatedly identified genes related to immune and synaptic function within risk loci for ASD [22,34].

Much of the strongest genetic evidence linking the complement system to aberrant synaptic pruning originates from schizophrenia research, in which structural variation in the complement component 4 (*C4*) locus and increased expression of the *C4A* isoform have been associated with disease risk and excessive synaptic elimination [18]. Although these findings provide an important mechanistic framework for studying complement-mediated pruning, they should not be directly extrapolated to ASD. Polymorphisms and variants in other complement-related genes, including *C1q*, *C3*, CR3/*ITGAM*, and their regulators, have been investigated in relation to ASD, suggesting that alterations in complement signaling may contribute to atypical synaptic refinement in specific genetic or biological contexts [22,35].

It is essential to distinguish the evidence for complement-mediated synaptic pruning in ASD from that established in schizophrenia. In schizophrenia, strong genetic and experimental evidence implicates increased *C4A* expression and complement-mediated synaptic elimination [18]. In ASD, by contrast, the available evidence is more heterogeneous and does not establish an equivalent *C4A*-driven mechanism. Alterations in complement components and other microglia-related pathways have been reported or proposed in ASD [36,37,38,39], but their relationship to the direction and extent of synaptic pruning remains incompletely defined. These observations suggest that complement-related mechanisms may differ across neurodevelopmental and psychiatric conditions and should therefore be interpreted within their specific genetic, developmental, and experimental contexts.

Several high-risk genes of microglial function for ASD are enriched in microglia and regulate their function. For example, variants in *TREM2* (triggering receptor expressed on myeloid cells 2), crucial for phagocytosis of phosphatidylserine tagged synapses, have been associated with an increased risk of ASD [23]. Similarly, genes such as *PTEN* and *SHANK3* mutated have been found to alter not only synaptic structure, but also neuron microglia interaction, which may affect surveillance and synaptic pruning.

ASD is a group of neurodevelopmental disorders with complex biology. Studies in functional genomics and mechanistic research in ASD have aimed to evaluate the role of risk genes in neurons and neuronal progenitor cells. However, the role of these ASD risk genes in other cell types has not been largely characterized. *ADNP*, a high-risk gene for ASD that modifies synaptic pruning, has been shown to act not only in neurons, but also intrinsically in microglia, which opens up a new perspective on altered synaptic pruning in autism pathogenesis and paves the way for precision medicine [40].

Therapeutic options for ASD are still limited. Activation of the classic complement system, an innate component of the immune signaling pathway, supports microglia-mediated synaptic pruning during development and disease. In particular, CD47, a ‘don’t eat me’ signal, protects synapses from inappropriate clearance. Jun and colleagues in 2025, investigating the role of CD47 in microglial phagocytosis in a mouse model of 16p11.2 deletion, observed that reducing CD47 signaling enhances microglia-mediated synaptic phagocytosis in the prefrontal cortex, followed by improved synaptic function and social interaction and behavior deficits. From a clinical standpoint, these experiments provide mechanistic insights into the role of CD47 as a basis for more effective autism treatment [41].

This genetic convergence of immune and synaptic pathways strongly suggests that risk variants may exert their effect, in part, by disturbing the neuroimmune interaction necessary for precise brain circuit development [11,34].

It is important to emphasize that the strength and nature of genetic evidence vary considerably across the genes discussed in this review. For some genes, such as *SHANK3* and *PTEN*, mutations have been directly associated with syndromic forms of ASD in human populations. For others, such as *C4A*, the most robust genetic and functional evidence for complement-mediated synaptic pruning comes from schizophrenia research, and its relevance to ASD remains primarily hypothetical, based on mechanistic overlap rather than direct genetic association in ASD cohorts. Similarly, evidence for *TREM2*, *ADNP*, and *SETDB1* in ASD derives largely from animal models, in vitro studies, or recent genetic screening approaches, and requires further validation in human ASD populations. Therefore, while the genetic convergence of immune and synaptic pathways provides a compelling framework for understanding ASD pathophysiology, the specific contribution of each gene and pathway to ASD risk should be interpreted within its appropriate level of evidence, distinguishing direct human genetic findings from preclinical or extrapolated data (Table 1).

### 5.2. In Vivo and Post-Mortem Findings Related to Synaptic Pruning in ASD

Direct studies of brain tissue providing tangible evidence of neuroimmune dysregulation in ASD have been reported by several research groups [11,24,34,42].

With regard to post-mortem studies, studies in individuals with ASD based on transcriptome analysis of prefrontal and temporal cortex reveal gene expression profiles consistent with immune and microglial activation at that level [11,34]. There is reported upregulation of genes involved in the innate immune response, antigen presentation, and neuroinflammation [11]. Immunohistochemically, multiple other studies confirm increased density and an activated state of microglia and astrocytes in key regions such as the prefrontal cortex and cerebellum [19,20]. Collectively, these findings indicate that a persistent pro-inflammatory brain microenvironment, incompatible with normal homeostatic microglial function, underlies precise synaptic pruning [20].

Studies from animal models were also based on environmental risk factors. Therefore, maternal immune activation (MIA), robustly reproducing ASD-like behaviors in offspring, demonstrated lasting microglial dysfunction [24]. Progeny of MIA mouse models show deficient synaptic pruning, particularly in the prefrontal cortex, and a hyperconnectivity phenotype [43]. Crucially, restoration of normal microglial function in these models can rescue both synaptic and behavioral deficits [42]. On the other hand, immunogenetic models such as mice overexpressing the human *C4A* gene develop excessive synaptic loss and deficits in working memory and sensorimotor gating [9]. This demonstrates the spectrum of behavioral consequences that can arise from unbalanced synaptic pruning.

### 5.3. Connectivity Evidence from Neuroimaging Techniques

A finding that is often interpreted in the context of altered synaptic pruning is the change in the architecture of brain networks, which is detectable by neuroimaging techniques.

Local Hyperconnectivity vs. Long-Range Hypoconnectivity: A relatively consistent finding in functional magnetic resonance imaging (fMRI) studies in ASD is the pattern of local hyperconnectivity (within the same brain region) together with hypoconnectivity or inefficient connectivity between distant regions (e.g., between frontal and temporal lobes) [34]. This pattern is compatible with the hypothesis of insufficient synaptic pruning at the local circuit level, which would leave an excess of redundant and ‘noisy’ connections, potentially hindering efficient information integration between specialized brain systems [44,45].

The regions where these connectivity anomalies are most consistently observed—the prefrontal cortex (social cognition, executive functions), the insular cortex (interoception, empathy), and the cerebellum (sensorimotor and cognitive processing)—are also those that show the most marked immunological and microglial alterations in post mortem studies [19,20,44]. This anatomical correlation is consistent with, but does not by itself demonstrate, a causal relationship between neuroimmune alterations and connectivity changes.

If neuroinflammation is a primary cause or a secondary consequence of early synaptic pathology has been a topic of several research group in studies with animals and humans [46,47]. The process can begin with early MIA or with a synaptic genetic vulnerability [46]. On the other side, glial activation in ASD may occur to activate microglia and astrocytes, which shift to a pro-inflammatory state [47]. This activated glia releases cytokines and reactive oxygen species that damage synapses. Synaptic damage, in turn, perpetuates glial activation, creating an amplification loop that leads to chronic neuroinflammation. This definition follow the concept of the peripheral-to-central inflammatory cascade-amplification model, where the neuroinflammation follows a cascade from the periphery to the CNS, with central amplification [25].

Together, the evidence from genes to brain circuits is consistent with a model in which genetic and epigenetic risk factors may influence the immunological mechanisms related to synaptic pruning. Such alterations could contribute to an abnormal organization of brain cytoarchitecture and connectivity, which may in turn be associated with deficits in social information integration, sensory sensitivity, and cognitive flexibility that characterize ASD. However, direct evidence establishing this sequence in living humans remains limited.

### 5.4. A Context-Dependent Framework for Synaptic Pruning Abnormalities in ASD

The evidence reviewed above supports a context-dependent model in which the direction and extent of synaptic pruning abnormalities—and their downstream effects on brain connectivity—are determined by multiple interacting factors. First, both insufficient and excessive synaptic elimination have been documented in ASD, depending on the genetic, developmental, and regional context, into other main aspects. Insufficient pruning leads to local hyperconnectivity, while excessive pruning promotes long-range hypoconnectivity; these patterns are not mutually exclusive and may coexist within the same individual across different brain regions. Second, pruning is a temporally and spatially regulated process, and alterations in the prefrontal cortex during adolescent pruning windows may yield different connectivity outcomes than disruptions in the cerebellum during early postnatal development, meaning that this regional and temporal heterogeneity must be incorporated into any comprehensive model [48]. Third, distinct ASD-associated genes affect pruning through different mechanisms: genes encoding synaptic proteins such as *SHANK3*, *PTEN*, and *NLGN3*/*4* tend to produce pruning deficits and hyperconnectivity, whereas genes regulating immune function such as *C4A*, *TREM2*, and *SETDB1* may drive excessive pruning and synaptic loss, so the genetic architecture of ASD predicts divergent connectivity phenotypes [49,50]. Fourth, in syndromic forms such as Fragile X, tuberous sclerosis, and Rett syndrome, specific genetic etiologies often produce characteristic connectivity patterns, whereas in idiopathic ASD, the absence of a defined monogenic cause yields more heterogeneous phenotypes, likely reflecting polygenic and environmental interactions [51]. Fifth, in some ASD cases, neuroinflammation and microglial activation may be primary drivers of synaptic pathology; in others, they may arise as secondary responses to synaptic dysfunction or even represent compensatory attempts to restore homeostasis, a distinction that has critical implications for therapeutic targeting. Sixth, not all individuals with ASD show detectable abnormalities in neuroimmune function or synaptic pruning, suggesting that additional mechanisms such as altered myelination, neurotransmitter imbalance, or intrinsic neuronal excitability can also produce ASD phenotypes independently of pruning dysregulation [52]. In general, these last considerations argue a not single unifying mechanism and support a multi-pathway, context-sensitive framework in which hyperconnectivity and hypoconnectivity represent different outcomes of distinct etiological trajectories converging on the common clinical phenotype of ASD [51].

## 6. Translational and Therapeutic Implications

The pathophysiological model that places neuroimmune dysregulation and aberrant synaptic pruning as central elements in ASD not only provides a mechanistic explanation but also a novel and promising avenue toward the discovery of new therapeutic targets in this entity. At the same time, emerging strategies seek to rebalance this axis, either by directly modulating microglial and complement function in the CNS or by addressing the systemic inflammatory factors that alter them [45,53]. This approach introduces a future shift from purely symptomatic interventions toward potentially disease-modifying therapies. Table 2 shows the preclinical evidence of the current clinical therapies, indicating if the studies have been conducted on animal models or human trials.

### 6.1. Intervention Strategies on Synaptic Pruning Effectors

Direct intervention on the cellular and molecular effectors involved in synaptic pruning offers the most direct route to correct circuit refinement defects. Some of these strategies include:

#### 6.1.1. Modulation of Microglial Function: This Strategy Presupposes Not Generalized Suppression but Rather “Re-Education” of Microglia Toward a Homeostatic and Phagocytically Competent Phenotype

- CSF1R Inhibition: Colony stimulating factor 1 receptor (CSF1R) is essential for microglial survival and proliferation, whereas CSF1R inhibitors (e.g., PLX3397) allow transient and controlled microglial depletion, followed by re-population with cells that may “reset” to a more functional state. In mouse models with microglial dysfunction, this approach has improved behavioral deficits [54].
- Nucleotide Receptor Modulators: Purinergic signaling through receptors such as P2RY12 (crucial for microglial surveillance) is altered in ASD models. In this regard, drugs that modulate this pathway could restore the motility and responsiveness of microglial processes to synaptic signals [55].
- *TREM2* Receptor Agonists: *TREM2* is a key receptor for phagocytosis of phosphatidylserine tagged synapses. Potentiating the *TREM2* signaling pathway could improve the precise elimination of redundant connections in cases of insufficient pruning [56].

#### 6.1.2. Complement System Inhibition: Given the Evidence for Complement Involvement in Aberrant Pruning, Its Pharmacological Inhibition Could Be a High Interest Strategy

- Anti *C1q* Antibodies: Compounds such as ANX005 (a human anti *C1q* monoclonal antibody) are designed to block the initial step of the classical pathway. They are already clinical trials for neurodegenerative diseases (such as Guillain–Barré and Alzheimer’s disease) where complement activation is pathological, paving the way for their possible use in neurodevelopmental disorders [57].
- Alternative or Terminal Pathway Inhibitors: Drugs that block *C3* convertase (e.g., pegcetacoplan) or the C5a anaphylatoxin receptor (C5aR1) could be used to attenuate excessive or chronic complement activation in the CNS [30].

### 6.2. Systemic Strategies and/or Peripheral Immunomodulation

Given the strong link with systemic inflammation, interventions acting outside the CNS are a logical complement.

#### Systemic Pharmacological Immunomodulation

- Cytokine Antagonists: In cases with clear biomarkers of elevated inflammation, biologic drugs already approved for autoimmune conditions (e.g., IL 6 antagonists such as tocilizumab or TNF α inhibitors) could be considered in tightly controlled clinical trials for specific ASD subgroups [58].
- Intravenous Immunoglobulins (IVIG): Used in autoimmune disorders, IVIG have broad immunomodulatory effects (neutralization of autoantibodies, modulation of cytokines). Their use in ASD has been associated with improvements in some patients, particularly those with evidence of concomitant autoimmunity [59].

### 6.3. Precision Medicine

The heterogeneity of ASD demands a shift toward precision medicine, which could be approached from different angles, such as stratification by immunological endophenotypes: the future of therapy in ASD could depend on the identification of peripheral biomarkers (cytokine profiles, autoantibodies, microbial signatures) that define more homogeneous patient subgroups (“immunotype 1”, “immunotype 2”). This would allow assigning specific therapies (e.g., a complement inhibitor or a particular probiotic) to the subgroup most likely to respond [60,61].

In addition, therapeutic windows of opportunity are also given information on the most active synaptic pruning mechanisms occurring during critical periods of postnatal development and adolescence, and interventions might be more effective if are applied early. However, brain plasticity in adulthood also suggests that neuroimmune modulation could have benefits at later stages [62]. The use of more rigorous clinical trials offering more promising strategies validated in randomized, double blind, controlled trials and with optimal biomarkers and secondary endpoints is also imperative. Others challenges include defining appropriate doses, treatment windows, and well-characterized patient subgroups [63].

Taken together, these therapeutic approaches remain largely exploratory in ASD. Direct clinical evidence supporting interventions targeting microglial function, complement signaling, or immune-mediated synaptic pruning is currently limited, whereas much of the mechanistic and therapeutic rationale derives from animal models, preclinical studies, or clinical experience in other neurological and immune-mediated conditions. Although these findings provide a rationale for further investigation, differences in ASD etiology, neuroimmune profiles, developmental stage, and the direction of potential pruning abnormalities may substantially influence treatment response. Therefore, modulation of neuroimmune and synaptic pruning pathways should currently be regarded as a promising research direction rather than an established disease-modifying therapeutic strategy for ASD. Well-designed clinical trials with appropriate biomarkers and biologically defined patient subgroups will be necessary to determine efficacy, safety, and optimal therapeutic windows.

## 7. Limitations

Although growing evidence supports the involvement of neuroimmune dysregulation in the ASD pathophysiology, several limitations should be considered when interpreting the available literature. ASD is a highly heterogeneous neurodevelopmental condition, encompassing individuals with substantial differences in clinical presentation, cognitive functioning, language abilities, comorbidities, and underlying biological characteristics. This complexity makes it unlikely that a single neuroimmune mechanism can explain the full spectrum of the disorder and may contribute to the variability observed across published studies. Another important challenge concerns clinical studies involving individuals with ASD, particularly in pediatric populations. Participant recruitment is often challenging because of ethical considerations, the need for parental consent, and the practical challenges associated with repeated clinical assessments, neuroimaging procedures, or biological sample collection. Consequently, many studies include relatively small and selected cohorts, limiting the statistical power and the generalizability of their findings. Furthermore, a considerable proportion of the current evidence is derived from experimental animal models and post-mortem human studies. While these approaches have provided valuable insights into the role of microglia, complement activation, and synaptic pruning, their findings cannot be directly extrapolated to the living human brain. An additional limitation concerns the terminology used to describe microglial and neuroimmune alterations. Terms such as neuroinflammation, microglial activation, reactive state, increased microglial density, and microglial dysfunction describe related but biologically distinct phenomena and should not be used interchangeably. In particular, evidence of increased microglial density or altered activation states does not, by itself, demonstrate impaired microglial function or defective synaptic pruning. Similarly, the presence of neuroinflammatory markers cannot be assumed to reflect a specific alteration in complement-mediated synapse elimination. This distinction is particularly important when integrating human post-mortem and neuroimaging findings with mechanistic evidence derived from experimental models. Future well-designed longitudinal studies integrating clinical, molecular, and neuroimaging data are needed to establish a better definition of the temporal relationship between neuroimmune alterations and ASD, and their impact on identifying reliable biomarkers and potential therapeutic targets.

## 8. Conclusions

Studies in ASD show the consolidation of a fundamental paradigm shift in their understanding. No longer seen solely as a disorder of purely synaptic or behavioral origin, ASD is increasingly conceptualized as a condition in which neuroimmune dysregulation, in the context of genetic and epigenetic factors, may contribute to aberrant synaptic pruning in a subset of individuals. The evolutionary co-option of immunological mechanisms, such as the complement system and microglial phagocytosis, to sculpt brain circuits during development provides an elegant and robust framework to explain how alterations in these processes could lead to the atypical connectivity that characterizes ASD. However, direct evidence establishing a causal sequence from neuroimmune dysregulation to altered pruning, connectivity changes, and clinical phenotype in living humans remains limited, and the heterogeneity of ASD suggests that multiple mechanisms are likely involved.

Although many questions remain to be answered, the framework proposed here provides guidelines for future research, emphasizing the need for longitudinal studies, improved biomarkers, and patient stratification to determine whether neuroimmune mechanisms represent primary drivers or modifiers of ASD pathophysiology. Deeper knowledge of the underlying mechanisms of brain immune communication could bring us closer to a future where the management of ASD could be more personalized, preventive, and fundamentally disease modifying.

## Figures and Tables

**Table 1:** Summary of the main ASD-associated genes discussed in this review, classified according to their primary functional role: those affecting synaptic architecture and those involved in immune regulation. This illustrates how diverse genetic pathways converge on common mechanisms of synaptic pruning and brain connectivity in ASD.

Category	Gene	Proposed Mechanism in ASD	Neuroimmune Function	Key Experimental Evidence
Synaptic architecture	*SHANK3*	Disruption of the postsynaptic scaffold alters the neuron-microglia interaction	It indirectly affects microglial function through altered synaptic signaling	*SHANK3* mutations cause Phelan McDermid syndrome with ASD; mouse models show pruning and connectivity defects
Synaptic architecture	*PTEN*	Hyperactivation of the mTOR pathway leading to altered synaptic density; affects microglial surveillance	It regulates cell growth and immune signaling; it modulates neuron-microglia communication	*PTEN* mutations cause macrocephaly and ASD; animal models show pruning deficits
Synaptic architecture	*NLGN3*/*NLGN4*	Alteration of synaptic adhesion; mutations destabilize the structure of synapses	Synaptic cell adhesion molecules; their dysfunction disrupts neuron-microglia communication	Mutations in neuroligins associated with ASD; animal models show alterations in synaptic transmission
Synaptic architecture	*NRXN1*	Alteration of presynaptic synaptic adhesion; interaction with neuroligins	Presynaptic synaptic cell adhesion molecule	Deletions and mutations of *NRXN1* associated with ASD; animal models show deficits in synaptic plasticity.
Synaptic architecture	*TSC1*/*TSC2*	Disruption of the mTOR pathway; synaptic hypertrophy and pruning disruption	mTORC1 negative regulators; affect synaptic signaling and microglial function	Mutations cause tuberous sclerosis with ASD; animal models show changes in synaptic density
Immune regulation	*C4A*	Excessive complement-mediated pruning (overexpression of *C4A* increases *C3* deposition)	Complement component; synapse tag for removal	Mouse models that overexpress human *C4A* show excessive synaptic loss and behavioral deficits GWAS have primarily linked *C4A* variants with schizophrenia risk; the evidence for *C4A* in ASD is more limited and heterogeneous, and direct extrapolation to ASD should be made with caution.
Immune regulation	*C1q*	Altered start of the classic complement cascade; possible under- or over-tagging	The complement activation starts; it opsonizes synapses	*C1q*-deficient mice show sustained synaptic connectivity; polymorphisms associated with ASD in genetic studies
Immune regulation	*C3*	Central effector of the complement; the deposition of *C3b* directs microglial phagocytosis	Opsonin; it binds to *CR3* in microglia	*C3* Mice/have *C3* pruning defects linked to ASD in immunogenetic cohorts
Immune regulation	*CR3*/*ITGAM*	Poor recognition of synapses tagged with *C3b*; defective microglial phagocytosis	Complement receptor 3; it mediates phagocytosis	CR3-deficient mice show hyperconnectivity; CR3 variants reported in ASD GWAS
Immune regulation	*TREM2*	Reduced phagocytosis of synapses with exposed phosphatidylserine; deficient microglial clearance	‘Eat-me’ signal receptor; promotes phagocytosis	*TREM2* variants associated with ASD; mouse models with VPA show decreased *TREM2* and synaptic changes
Immune regulation	*ADNP*	Alteration of microglial endocytosis and synaptic pruning	It modifies microglial synaptic pruning through endocytic trafficking	CRISPRi screen shows that loss of *ADNP* increases microglial motility and alters the proteome
Immune regulation	*SETDB1*	The deficiency raises C4b expression, triggering excessive pruning	Epigenetic regulator; modulates the expression of complement genes in neurons	*SETDB1* deficiency leads to excessive microglial pruning and autism-like behaviors

**Table 2:** Preclinical evidence of current therapy in clinical evaluation.

Therapy	Target/Mechanism	Evidence Type	Current Status
CSF1R inhibition (PLX3397)	Microglial depletion and repopulation	Animal models	Preclinical
Nucleotide receptor modulators (P2RY12)	Microglial surveillance and motility	Animal models; experimental	Preclinical
*TREM2* receptor agonists	Phagocytosis of phosphatidylserine-tagged synapses	Animal models	Preclinical
Anti-*C1q* antibodies (ANX005)	Classical complement pathway blockade	Clinical trials in Guillain-Barré, Huntington’s disease	Under clinical evaluation (for other indications)
Alternative/terminal pathway inhibitors (Pegcetacoplan, C5aR1 antagonists)	*C3* convertase or C5a receptor blockade	Preclinical; hypothetical in CNS	Preclinical
Cytokine antagonists (IL-6, TNF-α inhibitors)	Systemic immunomodulation	Proposed based on MIA models	Preclinical (no ASD trials)
Intravenous Immunoglobulins (IVIG)	Broad immunomodulation	Small pilot studies in ASD	Early-phase clinical observation

## Data Availability

No new data were created or analyzed in this study. Data sharing is not applicable to this article.

## Abbreviations

The following abbreviations are used in this manuscript:

ASD	Autism spectrum disorder
BBB	Blood–brain barrier
CNV	Copy number variation
CNS	Central nervous system
CR3	Complement receptor 3
CSF	Cerebrospinal fluid
CSF1R	Colony stimulating factor 1 receptor
fMRI	Functional magnetic resonance imaging
GWAS	Genome-wide association study
IL	Interleukin
MBP	Myelin basic protein
MIA	Maternal immune activation
NCAM	Neural cell adhesion molecule
SIRPα	Signal regulatory protein alpha
*TREM2*	Triggering receptor expressed on myeloid cells 2
VPA	Valproic acid

## References

[1] Hirota T., King B.H. (2023). Autism spectrum disorder: A review. JAMA.

[2] Exposito-Alonso D., Rico B. (2022). Mechanisms underlying circuit dysfunction in neurodevelopmental disorders. Annu. Rev. Genet..

[3] Salvador A.F.M., Abduljawad N., Kipnis J. (2024). Meningeal lymphatics in central nervous system diseases. Annu. Rev. Neurosci..

[4] Muzio L., Perego J. (2024). CNS resident innate immune cells: Guardians of CNS homeostasis. Int. J. Mol. Sci..

[5] Salvador A.F., de Lima K.A., Kipnis J. (2021). Neuromodulation by the immune system: A focus on cytokines. Nat. Rev. Immunol..

[6] Stevens B., Allen N.J., Vazquez L.E., Howell G.R., Christopherson K.S., Nouri N., Micheva K.D., Mehalow A.K., Huberman A.D., Stafford B. (2007). The classical complement cascade mediates CNS synapse elimination. Cell.

[7] Schafer D.P., Lehrman E.K., Kautzman A.G., Koyama R., Mardinly A.R., Yamasaki R., Ransohoff R.M., Greenberg M.E., Barres B.A., Stevens B. (2012). Microglia sculpt postnatal neural circuits in an activity and complement-dependent manner. Neuron.

[8] Paolicelli R.C., Sierra A., Stevens B., Tremblay M.-E., Aguzzi A., Ajami B., Amit I., Audinat E., Bechmann I., Bennett M. (2022). Microglia states and nomenclature: A field at its crossroads. Neuron.

[9] Yilmaz M., Yalcin E., Presumey J., Aw E., Ma M., Whelan C.W., Stevens B., McCarroll S.A., Carroll M.C. (2021). Overexpression of schizophrenia susceptibility factor human complement *C4A* promotes excessive synaptic loss and behavioral changes in mice. Nat. Neurosci..

[10] Guedes J.R., Ferreira P.A., Costa J., Laranjo M., Pinto M.J., Reis T., Cardoso A.M., Lebre C., Casquinha M., Gomes M. (2023). IL-4 shapes microglia-dependent pruning of the cerebellum during postnatal development. Neuron.

[11] Voineagu I., Wang X., Johnston P., Lowe J.K., Tian Y., Horvath S., Mill J., Cantor R.M., Blencowe B.J., Geschwind D.H. (2011). Transcriptomic analysis of autistic brain reveals convergent molecular pathology. Nature.

[12] Ilic N., Sarajlija A. (2025). Neuroglial dysregulation in autism Spectrum Disorder: Pathogenetic insights, genetic threads, and therapeutic horizons. Neuroglia.

[13] Aytulun A., Ünal D. (2026). Neuroinflammation and autism: What have we learned so far?. Int. J. Dev. Disabil..

[14] Lord C., Brugha T.S., Charman T., Cusack J., Dumas G., Frazier T., Jones E.J., Jones R.M., Pickles A., State M.W. (2020). Autism spectrum disorder. Nat. Rev. Dis. Primers.

[15] Zoghbi H.Y., Bear M.F. (2012). Synaptic dysfunction in neurodevelopmental disorders associated with autism and intellectual disabilities. Cold Spring Harb. Perspect. Biol..

[16] Louveau A., Smirnov I., Kipnis J. (2018). Structural and Functional Features of Central Nervous System Lymphatics. Nature.

[17] Paolicelli R.C., Bolasco G., Pagani F., Maggi L., Scianni M., Panzanelli P., Giustetto M., Ferreira T.A., Guiducci E., Dumas L. (2011). Synaptic pruning by microglia is necessary for normal brain development. Science.

[18] Sekar A., Bialas A.R., De Rivera H., Davis A., Hammond T.R., Kamitaki N., Tooley K., Presumey J., Baum M., Van Doren V. (2016). Schizophrenia risk from complex variation of complement component 4. Nature.

[19] Vargas D.L., Nascimbene C., Krishnan C., Zimmerman A.W., Pardo C.A. (2005). Neuroglial activation and neuroinflammation in the brain of patients with autism. Ann. Neurol. Off. J. Am. Neurol. Assoc. Child Neurol. Soc..

[20] Morgan J.T., Chana G., Pardo C.A., Achim C., Semendeferi K., Buckwalter J., Courchesne E., Everall I.P. (2010). Microglial activation and increased microglial density observed in the dorsolateral prefrontal cortex in autism. Biol. Psychiatry.

[21] Edmonson C., Ziats M.N., Rennert O.M. (2014). Altered glial marker expression in autistic post-mortem prefrontal cortex and cerebellum. Mol. Autism.

[22] Nudel R., Benros M.E., Krebs M.D., Allesøe R.L., Lemvigh C.K., Bybjerg-Grauholm J., Børglum A.D., Daly M.J., Nordentoft M., Mors O. (2019). Immunity and mental illness: Findings from a Danish population-based immunogenetic study of seven psychiatric and neurodevelopmental disorders. Eur. J. Hum. Genet..

[23] Estes M.L., McAllister A.K. (2016). Maternal immune activation: Implications for neuropsychiatric disorders. Science.

[24] Choi G.B., Yim Y.S., Wong H., Kim S., Kim H., Kim S.V., Hoeffer C.A., Littman D.R., Huh J.R. (2016). The maternal interleukin-17a pathway in mice promotes autism-like phenotypes in offspring. Science.

[25] Zhang X., Xue H., Zhu C., Ji C., Li Y., Liu W. (2026). Neuroinflammatory mechanisms and pharmacological advances in autism spectrum disorder: From inflammatory pathways to targeted interventions. Front. Immunol..

[26] Szabó D., Otrokocsi L., Sperlágh B. (2025). The role of maternal infections in neurodevelopmental psychiatric disorders: Focus on the P2X7/NLRP3/IL-1β signalling pathway. J. Neuroinflamm..

[27] Khan H., Wang Y.-M., Iftikhar I., Arif B., Khan B., Mubin Mustafa M., Al-hussain F., Bashir S., Li H.-T. (2026). Gut-Brain Axis Mechanisms and Microbiome Abnormalities in Autism Spectrum Disorder and Therapeutic Implications. Curr. Neuropharmacol..

[28] Petropoulos A., Stavropoulou E., Tsigalou C., Bezirtzoglou E. (2025). Microbiota gut–brain axis and autism spectrum disorder: Mechanisms and therapeutic perspectives. Nutrients.

[29] Stevens B., Johnson M.B. (2021). The complement cascade repurposed in the brain. Nat. Rev. Immunol..

[30] Gomez-Arboledas A., Acharya M.M., Tenner A.J. (2021). The role of complement in synaptic pruning and neurodegeneration. ImmunoTargets Ther..

[31] Scott-Hewitt N., Perrucci F., Morini R., Erreni M., Mahoney M., Witkowska A., Carey A., Faggiani E., Schuetz L.T., Mason S. (2020). Local externalization of phosphatidylserine mediates developmental synaptic pruning by microglia. EMBO J..

[32] Pagani M., Barsotti N., Bertero A., Trakoshis S., Ulysse L., Locarno A., Miseviciute I., De Felice A., Canella C., Supekar K. (2021). mTOR-related synaptic pathology causes autism spectrum disorder-associated functional hyperconnectivity. Nat. Commun..

[33] Monet M.C., Quan N. (2023). Complex neuroimmune involvement in neurodevelopment: A mini-review. J. Inflamm. Res..

[34] Gandal M.J., Zhang P., Hadjimichael E., Walker R.L., Chen C., Liu S., Won H., Van Bakel H., Varghese M., Wang Y. (2018). Transcriptome-wide isoform-level dysregulation in ASD, schizophrenia, and bipolar disorder. Science.

[35] Soteros B.M., Sia G.M. (2022). Complement and microglia dependent synapse elimination in brain development. WIREs Mech. Dis..

[36] Gunaydin M., Dogan O., Gunay F., Cikili-Uytun M., Celik-Buyukceran Ö., Oztop D.B. (2025). Complement system dysfunction in autism spectrum disorder: Evidence for altered *C1q* and *C3* levels (complement system dysfunction in ASD). Acta Neuropsychiatr..

[37] Willem H. (2026). The Dual Role of Microglial Synaptic Pruning in Autism Spectrum Disorder and Schizophrenia: A Literature Review. Undergrad. Res. Nat. Clin. Sci. Technol. J..

[38] Veremeyko T., Jiang R., He M., Ponomarev E.D. (2023). Complement C4-deficient mice have a high mortality rate during PTZ-induced epileptic seizures, which correlates with cognitive problems and the deficiency in the expression of Egr1 and other immediate early genes. Front. Cell. Neurosci..

[39] Sierra D.P., Tripathi A., Pillai A. (2022). Dysregulation of complement system in neuropsychiatric disorders: A mini review. Biomark. Neuropsychiatry.

[40] Teter O.M., McQuade A., Hagan V., Liang W., Dräger N.M., Sattler S.M., Holmes B.B., Castillo V.C., Papakis V., Leng K. (2025). CRISPRi-based screen of autism spectrum disorder risk genes in microglia uncovers roles of *ADNP* in microglia endocytosis and synaptic pruning. Mol. Psychiatry.

[41] Ju J., Pan Y., Yang X., Li X., Chen J., Wu S., Hou S.-T. (2025). The “don’t eat me” signal CD47 is associated with microglial phagocytosis defects and autism-like behaviors in 16p11. 2 deletion mice. Proc. Natl. Acad. Sci. USA.

[42] Zhan Y., Paolicelli R.C., Sforazzini F., Weinhard L., Bolasco G., Pagani F., Vyssotski A.L., Bifone A., Gozzi A., Ragozzino D. (2014). Deficient neuron-microglia signaling results in impaired functional brain connectivity and social behavior. Nat. Neurosci..

[43] Namvarpour Z., Azhdari S., Lotfi R., Pahang H., Omidi H., Namvarpour M., Amini A. (2025). Impact of mild prenatal LPS-induced inflammation and postnatal stress on synaptic plasticity and neuroimmune responses in the prefrontal cortex of adult male mice. Tissue Cell.

[44] Courchesne E., Campbell K., Solso S. (2011). Brain growth across the life span in autism: Age-specific changes in anatomical pathology. Brain Res..

[45] Eissa N., Sadeq A., Sasse A., Sadek B. (2020). Role of neuroinflammation in autism spectrum disorder and the emergence of brain histaminergic system. Lessons also for BPSD?. Front. Pharmacol..

[46] Qiao S.-N., Wang S.E., Kim K.-Y., Jo S., Jiang Y.-H. (2026). Inflammation increases the penetrance of behavioral impairment in Shank3 haploinsufficiency mice—Can it explain the behavioral regression in Autism?. Mol. Psychiatry.

[47] Schiano-Visconte M., Balasco L., Casarosa S., Bozzi Y. (2026). Microglia, cerebellar inflammation, and autism spectrum disorders: Developmental mechanisms and therapeutic perspectives. Neuroscience.

[48] Osterne V.J.S., Oliveira M.V., Pinto-Junior V.R., Mota F.S.B., Leal R.B., Cavada B.S., Nascimento K.S. (2026). Microglial State Mismatch in Autism Spectrum Disorder: Timing, Circuit Specificity and Glycan-Mediated Recognition. Neuroglia.

[49] Das M.R., Rahman M., Zhou C. (2025). Structural Insights into Protein Mutations Related to Autism Spectrum Disorders: A Systematic Review. ACS Chem. Neurosci..

[50] Chen S., Zhang B., Qin T., Zhu M., Chen Q., Xia L., Pan H., Yang Q., Guo S., Gong R. (2026). Endogenous retrovirus-derived RNA-DNA hybrids induce microglial synaptic pruning in autism models. Neuron.

[51] Carroll L., Braeutigam S., Dawes J.M., Krsnik Z., Kostovic I., Coutinho E., Dewing J.M., Horton C.A., Gomez-Nicola D., Menassa D.A. (2021). Autism spectrum disorders: Multiple routes to, and multiple consequences of, abnormal synaptic function and connectivity. Neurosci..

[52] Lian A., He M., Zhang H., Yang Y. (2026). Microglia in autism spectrum disorder: Heterogeneity, immunometabolism, and synapse-related pathways. Front. Immunol..

[53] Voineagu I., Eapen V. (2013). Converging pathways in autism spectrum disorders: Interplay between synaptic dysfunction and immune responses. Front. Hum. Neurosci..

[54] Elmore M.R., Najafi A.R., Koike M.A., Dagher N.N., Spangenberg E.E., Rice R.A., Kitazawa M., Matusow B., Nguyen H., West B.L. (2014). Colony-stimulating factor 1 receptor signaling is necessary for microglia viability, unmasking a microglia progenitor cell in the adult brain. Neuron.

[55] Badimon A., Strasburger H.J., Ayata P., Chen X., Nair A., Ikegami A., Hwang P., Chan A.T., Graves S.M., Uweru J.O. (2020). Negative feedback control of neuronal activity by microglia. Nature.

[56] Luo L., Chen J., Wu Q., Yuan B., Hu C., Yang T., Wei H., Li T. (2023). Prenatally VPA exposure is likely to cause autistic-like behavior in the rats offspring via *TREM2* down-regulation to affect the microglial activation and synapse alterations. Environ. Toxicol. Pharmacol..

[57] Mohammad Q.D., Islam Z., Papri N., Hayat S., Jahan I., Azad K.A.K., Artis D.R., Hoehn B., Humphriss E., Lin P. (2025). Results from a phase 1 study evaluating the safety, tolerability, pharmacokinetics, pharmacodynamics, and efficacy of ANX005, a *C1q* inhibitor, in patients with Guillain–Barré syndrome. J. Peripher. Nerv. Syst..

[58] Goines P.E., Ashwood P. (2013). Cytokine dysregulation in autism spectrum disorders (ASD): Possible role of the environment. Neurotoxicology Teratol..

[59] Melamed I.R., Heffron M., Testori A., Lipe K. (2018). A pilot study of high-dose intravenous immunoglobulin 5% for autism: Impact on autism spectrum and markers of neuroinflammation. Autism Res..

[60] Gesundheit B., Rosenzweig J.P., Naor D., Lerer B., Zachor D.A., Procházka V., Melamed M., Kristt D.A., Steinberg A., Shulman C. (2013). Immunological and autoimmune considerations of Autism Spectrum Disorders. J. Autoimmun..

[61] Hughes H.K., Mills Ko E., Rose D., Ashwood P. (2018). Immune dysfunction and autoimmunity as pathological mechanisms in autism spectrum disorders. Front. Cell. Neurosci..

[62] Pardo C.A., Vargas D.L., Zimmerman A.W. (2005). Immunity, neuroglia and neuroinflammation in autism. Int. Rev. Psychiatry.

[63] Lord C., Charman T., Havdahl A., Carbone P., Anagnostou E., Boyd B., Carr T., De Vries P.J., Dissanayake C., Divan G. (2022). The Lancet Commission on the future of care and clinical research in autism. Lancet.

